# Age at menopause is inversely related to the prevalence of common gynecologic cancers: a study based on NHANES

**DOI:** 10.3389/fendo.2023.1218045

**Published:** 2023-11-16

**Authors:** Guangdong Cheng, Mengmeng Wang, Hao Sun, Jingjiang Lai, Yukun Feng, Hongjin Liu, Yuwang Shang, Yuan Zhao, Bingli Zuo, Youhua Lu

**Affiliations:** ^1^ Department of Clinical Laboratory, Shandong Provincial Hospital Affiliated to Shandong First Medical University, Jinan, China; ^2^ Xiyuan Hospital, China Academy of Chinese Medical Sciences, Beijing, China; ^3^ Shandong Cancer Hospital and Institute, Shandong First Medical University and Shandong Academy of Medical Sciences, Jinan, China; ^4^ Innovative Institute of Chinese Medicine, Shandong University of Traditional Chinese 25 Medicine, Jinan, China

**Keywords:** retrospective study, menopause, cancer, gynecology, NHANES

## Abstract

**Background:**

The fluctuation or even loss of estrogen level caused by menopause in women, and most gynecological cancers often occur before and after menopause, so the age of menopause may be related to the occurrence of gynecological cancer.

**Aim:**

To investigate whether the age at menopause is independently associated with the incidence of gynecological cancers and to analyze the possible influencing factors.

**Methods:**

We selected the NHANES public database to conduct the study, and by excluding relevant influencing factors, we finally included 5706 NHANES participants who had full data on age at menopause and the occurrence of gynecologic cancers to analyze the relationship between the amount of age at menopause and gynecologic cancers based on univariate or multifactorial logistic regression analysis. Further, the relationship between age at menopause and the prevalence of different gynecologic cancers was investigated, and changes in the prevalence of different gynecologic cancers by age at menopause subgroups were observed. Finally, other relevant factors affecting the prevalence of gynecologic cancers were further investigated by subgroup analysis as well as subcluster analysis.

**Results:**

Univariate logistic regression analysis between age at menopause and gynecologic tumor prevalence revealed a negative association between age at menopause and the prevalence of common gynecologic cancers ovarian and cervical cancer, and after adjusting for the effects of covariates, a higher risk of gynecologic tumors was found with statistically significant differences at earlier age at menopause. The regression results showed a negative association between age at menopause and gynecologic cancer prevalence in cervical and ovarian cancer patients (P<0.01,P<0.01). Cervical cancer (OR: 0.91, 95% CI: 0.87,0.94) and ovarian cancer (OR: 0.90, 95% CI: 0.86, 0.95) were more prevalent among those with younger age at menopause.

**Conclusion:**

Age at menopause is negatively associated with the prevalence of cervical and ovarian cancers, and the earlier the age at menopause, the greater the risk of developing gynecological cancers.

## Introduction

It is estimated that about 1.3 million new gynaecological cancers are diagnosed worldwide each year. Among the global female cancer deaths in 2020, cervical cancer and ovarian cancer ranked 4th and 8th among the three major gynecological malignancies, accounting for 12.4% of female cancer deaths, while endometrial cancer did not enter the top ten (1, 2). With the great threat of malignant tumors of the female reproductive system (mainly ovarian cancer, endometrial cancer, and cervical cancer) to women’s life safety, the risk factors related to the incidence of these three common gynecological tumors have gradually attracted people’s attention.

In the clinic, ovarian cancer is a silent killer known for its difficult to find, difficult to treat and easy to recur in female gynecological cancers, although the incidence of ovarian cancer is low, accounting for only 3% of female cancers, but it is one of the highest mortality rates in gynecological cancers (3, 4). Some scholars believe that multiple ovulation ruptures and repair theoretically increase the chance of malignant mutations, which may be related to factors that affect ovulation such as early menopause, late menarche, and oral contraceptives (5). In a randomized controlled trial of dietary modification by the Women’s Health Initiative, postmenopausal women who passed a low-fat diet had a reduced risk of ovarian cancer (3).Cervical cancer is a preventable and treatable cancer, more than 70% of which is closely related to HPV human papillomavirus 16, 18 and other serotypes (6), HPV vaccination provides a scientific basis for the early prevention and treatment of cervical cancer (2). Cervical cancer is also known as the “disease of wealth disparity” due to significant differences in morbidity and mortality between low- and middle-income countries (LMICs) and relatively high-income countries (HICs), which are determined by resources, including age-appropriate screening, vaccination and necessary treatment. Endometrial cancer mostly occurs after menopause (2), which is positively correlated with life expectancy (7). Studies have shown that non-antagonistic estrogens, which are not counteracted by progestogens, are associated with an increased risk of cancer, and long-term exposure can lead to endometrial hyperplasia, which greatly increases the chances of atypical hyperplasia developing into type I endometrial cancer. Non-antagonistic estrogens can be caused by pure estrogen therapy, obesity, and anovulatory menstrual cycles, where the ovaries have small estrogen-secreting cells but do not secrete progesterone.

These studies have shown a correlation between the occurrence of these cancers and estrogen levels, and overall, postmenopausal women have lower blood estrogen levels and a relatively low risk of developing these cancers (8). Estrogen levels are highest in women during their lifetime, but as women age (usually between 45 and 55 years), the function of the ovaries gradually decreases, eventually stopping ovulation and producing estrogen, called menopause, during which estrogen secretion decreases (9, 10). As we all know, estrogen is a protective sex hormone, and the fluctuation or even loss of estrogen levels caused by menopause plays an important role in women’s cardiovascular system, osteoporosis and fat metabolism (11–13), so the relationship between menopausal age and the incidence of gynecological tumors has also attracted attention. In clinical practice, most cases of gynecologic cancer are diagnosed after menopause (14–16), which raises the question: is there an association between age at menopause and the incidence of gynecologic cancer? What is the connection between them, which has attracted the attention of women around the world, and it is becoming increasingly important to achieve universal screening for related cancers in perimenopausal and different menopausal age groups.

The NHANES database has a huge data resource, which includes Demog Data、Dietary Data、Examination Data、Laboratory Data、Questionnaire Data、Limited Access Data. Through this database, we can use it to determine the prevalence of major diseases and risk factors for diseases (17–19). Therefore, in this study, we aim to mine data through NHANES database and analyze the relationship between menopausal age and gynecological cancer based on univariate or multivariate logistic regression, which provides an important scientific basis for women’s health management.

## Materials and methods

### Study design and sample

**Figure 1:**
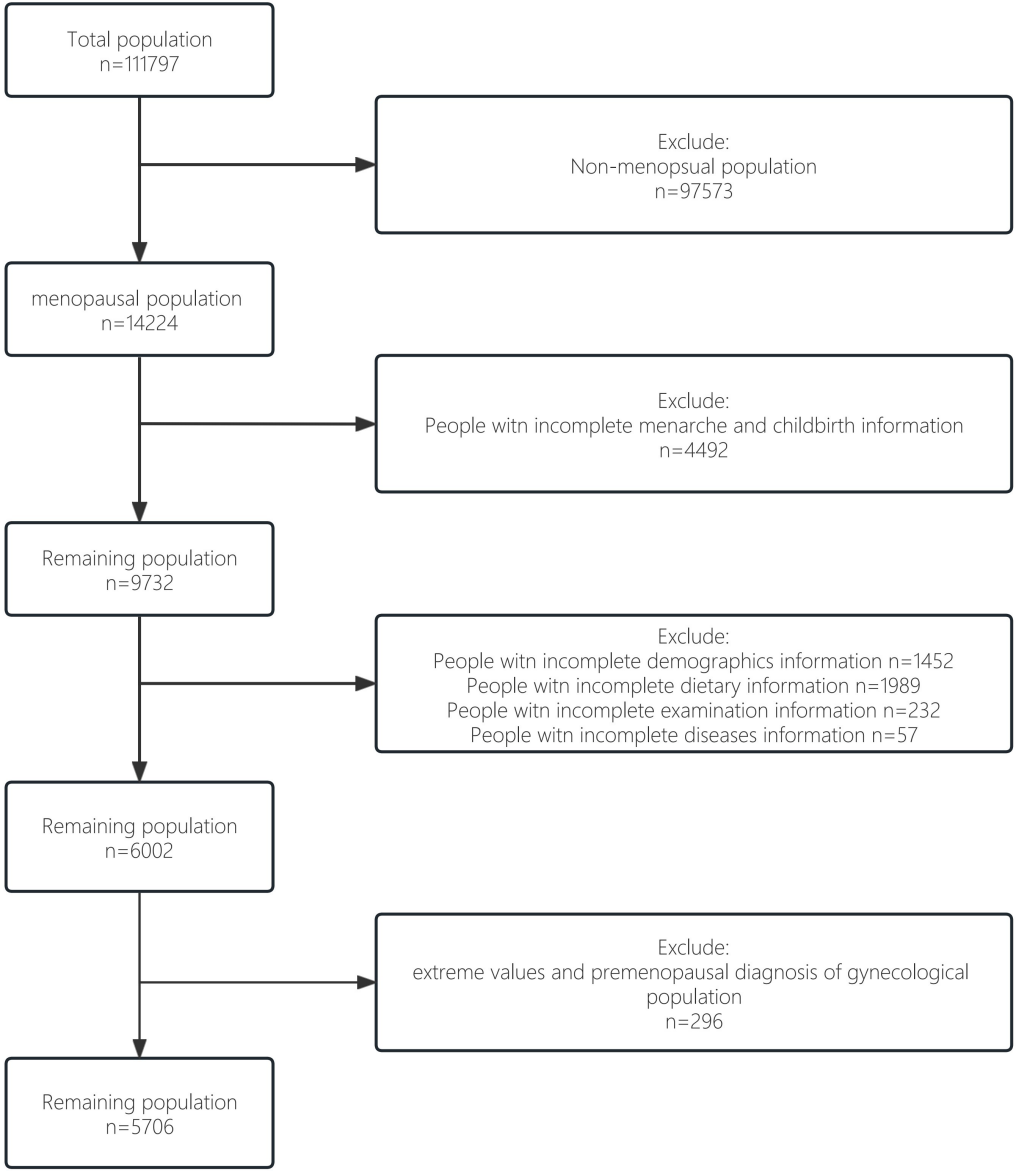
Flow chart of study sample screening.

We obtained data from the nationally representative National Health and Nutrition Examination Survey (NHANES) (20). The survey used a complex multi-stage probability sample representing the civilian population in all 50 states and the District of Columbia in the United States. In the current study, 5706 NHANES participants were ultimately included, representing 21950882 postmenopausal women in the United States, covering 14 years (2007-2020). The survey was approved by the Institutional Review Board of the National Center for Health Statistics and all patients had informed consent. The flow chart for selecting the study sample is shown in **Figure 1**. The total population (n=111797) was screened for the non-menopausal population (n=97573). Then among menopausal women, we excluded some groups with incomplete information on menarche and childbirth (n=4492). Of the remaining 9732 participants, groups without complete information on demographics, disease, diet and necessary testing were excluded (n=3730). Finally, to improve the scientific validity and reliability of the results, we excluded from the remaining population those extreme values and premenopausal diagnosis of gynecological population (n=296), resulting in our idealised study data population (n=5706).

### Sociodemographic characteristics

Sociodemographic characteristics include age, race, and educational attainment. Race includes non-Hispanic white, non-Hispanic black, Mexican-American, other Hispanic, other race, and education level includes less than high school, high school and high school and beyond.

### Nutritional status

To study the nutritional status of the population, we obtained data from the household property to income ratio (PIR) as well as the body mass index (BMI) and energy intake. In general, the higher the PIR, the more it is in terms of nutritional intake and physical work, and its BMI is also relatively higher than that of the low-income group. we divided the data according to the median and selected the household PIR, choosing 2.3% as the cut-off value. The BMI screening was divided into >25Kg/m^2^ or ≤25Kg/m^2^.

### Behavioral habits

We categorized behavioral habits by how often they smoked and drank, and participants were divided into: never (less than a hundred cigarettes smoked in a lifetime), before (smoked more). There are more than 100 cigarettes in life, now they don’t smoke at all), and now (more than 100 cigarettes are smoked. cigarettes in life, sometimes or every day). People who drink alcohol are divided into five groups: 1. Never drank less than 1 drink.) 2. Before (Drinking. In the past 12 months, women have drunk more than 1 drink and now do not drink at all), 3 mild (women drink ≥ 1 drink per day, ≥ drink 2 drinks per day. 4. Moderate (2 cups a day for women, 3 cups a day for ≥.), 5. Severe (women ≥ 3 cups a day, ≥ 4 cups a day.) (21)。 In addition, the energy intake of the population (kcal) is also a reference variable for behavioral habits. The energy intake is divided into < 1580KJ or ≥1580KJ.

### Underlying medical conditions

We included diabetes mellitus and hypertension, two underlying diseases associated with the development and progression of gynecologic cancers. The clinical diagnostic criteria for diabetes mellitus were.1. The doctor informs the diagnosis; 3. Fasting blood glucose ≥ 7.0 mmol/L 2. Glycohemoglobin HbA1c>6.5%;4. Random blood glucose≥ 11.1 mmol/L; 5. Two-hour OGTT blood glucose ≥ 11.1 mmol/L; 6. Already using diabetes medications or insulin. The clinical diagnostic criteria for hypertension is ≥140/90 mmHg. The average blood pressure standards are as follows:1. If only one blood pressure reading is obtained, the number is judged to be average; 2. If there is more than one blood pressure reading, remove the first reading each time. 3. If the diastolic blood pressure reading is zero, it is not used to calculate the average diastolic blood pressure; 4. If all diastolic blood pressure readings are zero, the average is zero.

### Statistical analysis

Data obtained from screening were analyzed using R (v.4.2.1). Statistical analysis of gynecologic cancer incidence by age, age at menopause, race, education, PIR, BMI, smoking, alcohol consumption levels, menarche, childbirth and presence of hypertension and diabetes was performed. Univariate and multivariate logistic regression analysis between different age at menopause and different gynecologic cancers. We included age, race, education level, BMI, PIR, smoking, alcohol consumption, energy intake, menarche, childbirth, hypertension, diabetes, and cancer in different regression models to obtain univariate and multivariate logistic regression statistical results and significance. In the study of the association between age at menopause onset and cervical, we also adjusted for age, race, education, BMI, PIR, smoking, alcohol consumption, energy intake, hypertension, and diabetes, and finally performed subgroup analyses for ovarian and cervical cancers.

## Results

The baseline characteristics of the study population are shown in **Figure 1**, where the overall weighted prevalence of gynecologic cancers was 3.69%. The results showed statistically significant differences in gynecologic cancer prevalence by age, race, education level, PIR, BMI, smoking, alcohol consumption level, energy intake, and presence of hypertension and diabetes mellitus. **Table 1**. Baseline characteristics of the study population.

**Table 1:** Participant characteristics (N = 5,706) in NHANES 2007–2020.

Characteristics	Gynecological Cancer		*p* value
Total	no	yes	
**Age~ years**	96.75(91.20,102.29)	3.25(2.59, 3.92)	
	61.68(61.24,62.11)	58.85(56.56,61.14)	0.02
**Race~%**			<0.01
Non-Hispanic White	76.95(74.66,79.25)	83.72(77.39,90.06)	
Non-Hispanic Black	10.01(8.59,11.44)	4.13(1.91, 6.34)	
Mexican American	4.56(3.54,5.58)	3.23(1.26,5.19)	
Other Hispanic	3.92(3.12,4.72)	4.16(1.28,7.04)	
Other Race	4.56(3.85,5.26)	4.76(0.74,8.79)	
**Education level~%**			0.19
Less than high school	15.64(14.14,17.15)	15.94(9.99,21.89)	
High school	26.26(24.69,27.84)	31.33(22.02,40.65)	
More than high school	58.09(56.02,60.16)	52.72(42.72,62.73)	
**Family PIR**	3.11(3.02,3.19)	2.94(2.64,3.25)	0.29
**BMI~ kg/m2**	29.73(29.43,30.02)	31.15(29.67,32.62)	0.04
**Smoking behavior~%**			<0.01
never	57.57(55.74,59.41)	43.15(34.10,52.20)	
former	27.62(25.84,29.39)	27.57(18.91,36.23)	
now	14.81(13.29,16.33)	29.28(20.15,38.41)	
**Alcohol consumption~%**			0.01
never	15.70(14.27,17.12)	8.56(4.26,12.86)	
former	18.03(16.69,19.37)	23.18(15.90,30.46)	
mild	38.35(36.45,40.25)	31.20(22.63,39.77)	
moderate	19.19(17.63,20.75)	19.52(9.97,29.07)	
heavy	8.73(7.65, 9.82)	17.54(8.14,26.95)	
**Energy intake~kcal**	1696.78(1673.56,1720.00)	1717.79(1609.63,1825.95)	0.70
**Hypertension~%**			1.00
yes	58.07(56.50,59.64)	61.69(51.40,71.98)	
no	41.93(40.36,43.50)	38.31(28.02,48.60)	
**Diabetes~%**			0.93
yes	20.73(19.27,22.19)	18.95(12.43,25.47)	
no	79.27(77.81,80.73)	81.05(74.53,87.57)	
**Menopause~years**	45.21(44.94,45.49)	40.00(37.84,42.16)	<0.01
**First menstruation~years**	12.72(12.66,12.78)	12.41(12.07,12.75)	0.10
**Livng birth**	2.67(2.60,2.74)	2.60(2.34,2.86)	0.62

OR, odds ratio; CI, confidence interval; PIR, property income ratio; BMI, body mass index; Data are shown as mean (x ®)or n (%) and presented incorporating sample weights or prevalence (%) and 95%CI.

### Relationship between age of menopause and the prevalence of different gynaecological cancers

**Figure 2:**
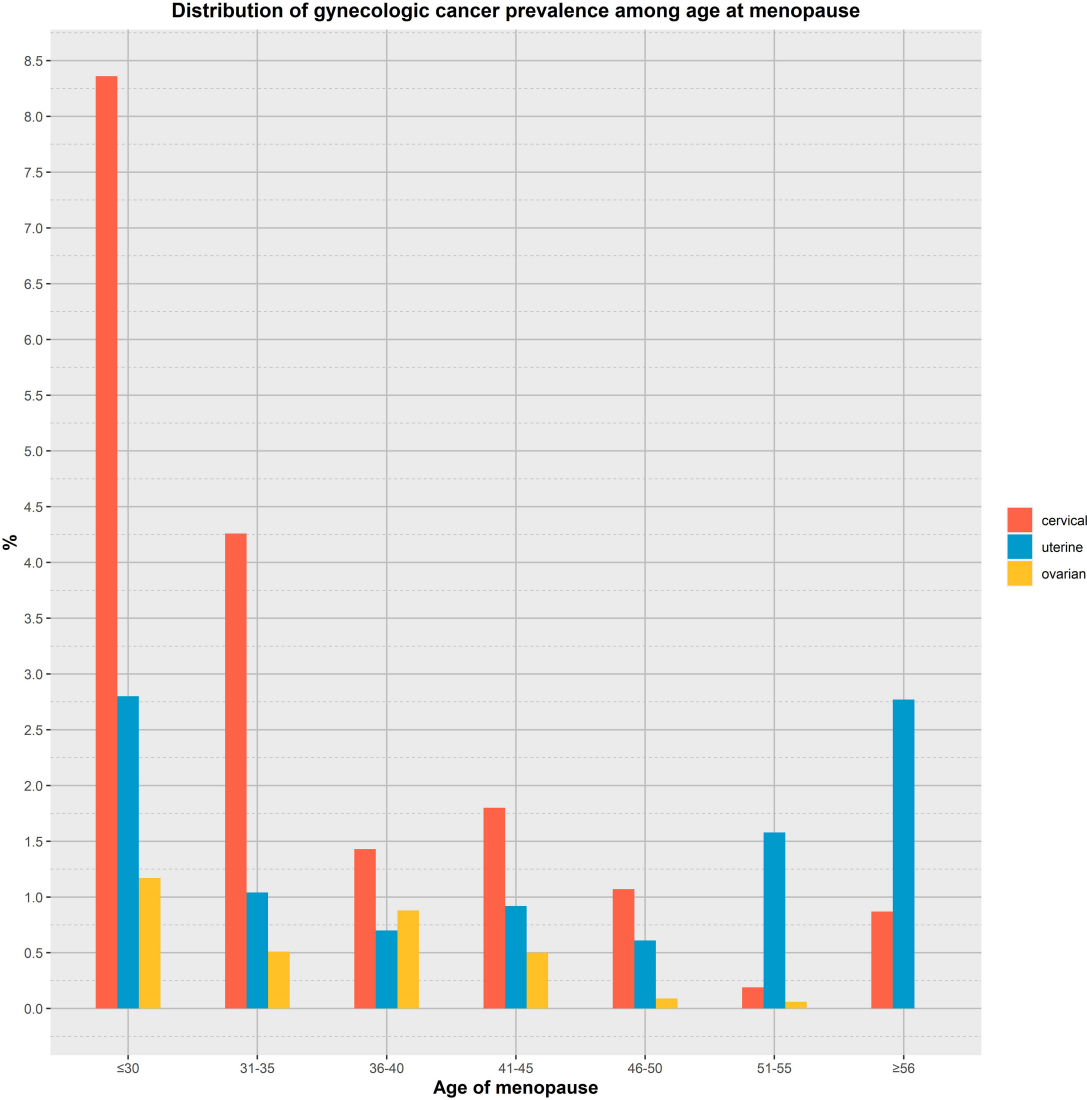
Analyzing the relationship between age at menopause and the prevalence of gynaecological cancer by age group.

The results of univariate and multivariate logistic regression analysis between the onset of menopausal age and gynecologic cancers are shown in **Table 2**. Among them, There was an inverse association between age at menopause and the prevalence of gynaecological cancer (OR: 0.93, 95% CI: 0.90,0.96), and the difference was statistically significant (P<0.01). Model 1 was adjusted for age and race, and the results showed that there was an inverse association between menopausal age and the prevalence of gynaecological cancer (OR: 0.93, 95% CI: 0.90-0.96), and the difference was statistically significant (P<0.01). Model 2, adjusted for age, race, first menstruation age and living birth, showed an inverse association between age at menopause and gynecologic cancer (OR: 0.93, 95% CI: 0.90-0.96), with statistically significant differences (P <0.01).Model 3 was adjusted for age, race, first menstruation age, living birth, education, BMI, PIR, smoking, alcohol consumption level, energy intake, and showed that there was an inverse association between age at menopause and the prevalence of gynecologic cancer (OR: 0.93, 95% CI: 0.90-0.96), and the difference was statistically significant (P <0.01). Model 4 was adjusted for a age, race, first menstruation age, living birth, education, BMI, PIR, smoking, alcohol consumption level, energy intake, hypertension and diabetes, and showed that there was an inverse association between age at menopause and the prevalence of gynecologic cancer (OR: 0.93, 95% CI: 0.90-0.96), and the difference was statistically significant (P <0.01).The global mean age of women at overall natural menopause was 48.8 years (95% CI 48.3,49.2) and the average age of menopause for women in the United States was 51 years (22). Therefore, we divided into 7 groups according to different menopausal ages, as shown in **Figure 2**, respectively, observing the relationship between menopausal age less than 30 years, between 30 and 35 years old, 36 to 40 years old, 41 to 45 years old, 46 to 50 years old, 51 to 55 years old, and greater than or equal to 56 years old and the occurrence of gynecological cancer, the results showed that the incidence of menopausal women before the age of 35 was greatly increased, and the difference was statistically significant.

**Table 2:** Odds ratios (OR), 95% CI, and P values for univariate and multivariate logistic regression analysis between gynecologic cancer and menopause.

outcomes	model	OR(95%CI)	*P* value
**Gynecological Cancer**	Crude	0.93(0.90,0.96)	<0.01
	Model1	0.93(0.90,0.96)	<0.01
	Model2	0.93(0.90,0.96)	<0.01
	Model3	0.93(0.90,0.96)	<0.01
	Model4	0.93(0.90,0.96)	<0.01

Crude is an unadjusted model, model 1 is a model adjusted for age and race, model 2 is a model adjusted for age, race, first menstruation age and living birth. model 3 is a model adjusted for age, race, first menstruation age, living birth, education, BMI, PIR, smoking, alcohol consumption level, energy intake. model 4 is a model adjusted for age, race, first menstruation age, living birth, education, BMI, PIR, smoking, alcohol consumption level, energy intake, hypertension and diabetes.

### Relationship between menopause and incidence of gynaecological cancer

**Figure 3:**
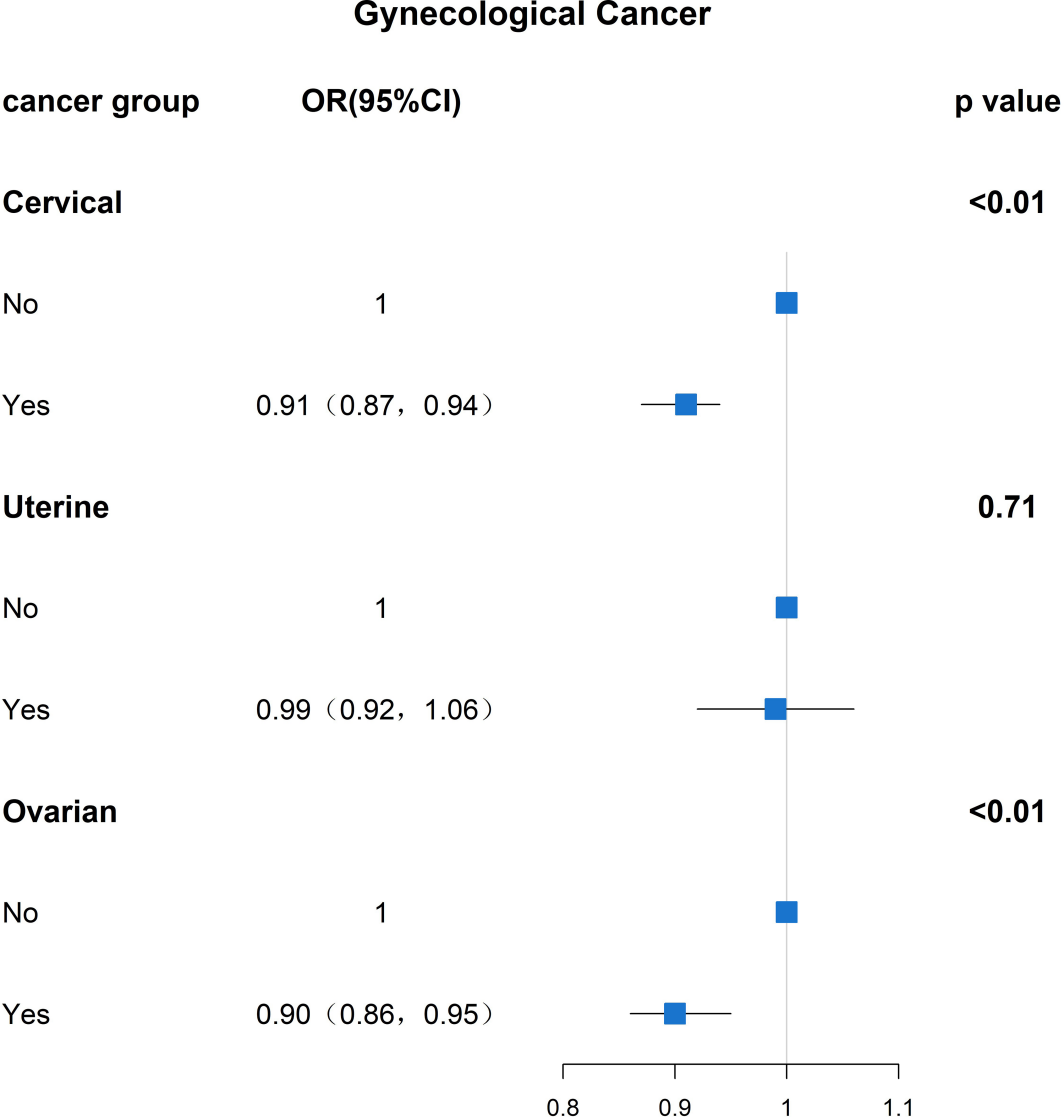
Results analyzed between age at menopause and prevalence of gynaecological cancer (OR(95% CI) and P value).

To investigate whether menopause is associated with gynecologic age, we performed a regression analysis between menopause and the incidence of three major gynecologic cancers, as shown in **Figure 3** for the relationship between age at menopause and the incidence of different gynecologic cancers. After adjusting for age, race, first menstruation age, living birth, education, BMI, PIR, smoking, alcohol consumption level, energy intake, hypertension and diabetes, the regression results revealed that age at menopause was inversely associated with the prevalence of gynecologic cancers in patients with cervical and ovarian cancer (P<0. 01, P<0.01). People of younger menopausal age are more likely to develop cervical cancer (OR: 0.91, 95% CI: 0.87,0.94) and ovarian cancer (OR: 0.90, 95% CI: 0.86,0.95).

### Subgroup analysis

The results of the subgroup analysis of gynecologic cancers are shown in **Table 3**, The results showed that people aged 65(P<0.01) and above with high school education or below(P<0.01), non-Hispanic whites(P<0.01), non-Hispanic blacks(P=0.04), and other Hispanics(P<0.01), had drunk alcohol and still drank alcohol(P=0.01, P=0.02, P=0.01, P=0.01). The prevalence of gynaecological cancer was higher in the population and the prevalence of gynaecological cancer is higher in people with younger menopause age than in people with older menopause.

**Table 3:** Odds ratios (OR), 95% CI, and P values for univariate and multivariate logistic regression analysis between gynaecological cancer and menopausal age.

Subgroup Variable	Gynecological Cancer OR(95%CI)	*P* value
Age		
<65	0.92(0.88,0.95)	<0.01
≥65	0.96(0.90,1.02)	0.17
Race		
Non-Hispanic White	0.93(0.89,0.96)	<0.01
Non-Hispanic Black	0.93(0.97,1.00)	0.04
Mexican American	0.95(0.87,1.04)	0.28
Other Hispanic	0.89(0.82,0.96)	<0.01
Other Race	1.00(0.90,1.12)	0.96
Education level		
Less than high school	0.90(0.85,0.95)	<0.01
High school	0.95(0.89,1.01)	0.10
More than high school	0.92(0.89,0.96)	<0.01
Family PIR		
<2.3	0.93(0.90,0.97)	<0.01
≥ 2.3	0.93(0.89,0.98)	<0.01
BMI		
<25	0.90(0.86,0.95)	<0.01
≥25	0.94(0.90,0.97)	<0.01
Smoking behavior		
never	0.93(0.89,0.97)	<0.01
former	0.92(0.87,0.98)	0.01
now	0.94(0.88,1.00)	0.05
Alcohol consumption		
never	0.97(0.90,1.05)	0.47
former	0.93(0.88,0.98)	0.01
mild	0.94(0.89,0.99)	0.02
moderate	0.90(0.84,0.97)	0.01
heavy	0.91(0.85,0.98)	0.01
Energy intake		
<1580	0.91(0.86,0.96)	<0.01
≥1580	0.94(0.91,0.98)	<0.01
Hypertension		
yes	0.93(0.90,0.97)	<0.01
no	0.92(0.88,0.97)	<0.01
Diabetes		
yes	0.94(0.89,1.00)	0.04
no	0.93(0.89,0.96)	<0.01

PIR, energy intake is divided into groups according to median;model is adjusted for age, race, first menstruation age, living birth, education, BMI, PIR, smoking, alcohol consumption level, energy intake, hypertension and diabetes.

The results of the subgroup analysis of cervical cancer are described in **Table 4**, in non-Hispanic whites(P<0.01), non-Hispanic blacks (P=0.01), with high school education or above(P<0.01), who are still drinking alcohol, (P=0.01, P<0.01, P<0.01), age at menopause was inversely correlated with cervical cancer prevalence. The results of the subgroup analysis of ovarian cancer are shown in **Table 5**, in people under 65 years of age(P<0.01), non-Hispanic whites(P<0.01), Mexican(P<0.01), other Hispanic(P<0.01), other people(P<0.01), never smoked(P<0.01), former smoking(P=0.03), had ever drunk, had consumed alcohol mild and light(P<0.01, P<0.01, P=0.03), had diabetes(P<0.01), the age of menopause was inversely correlated with the prevalence of ovarian cancer.

**Table 4:** Odds ratio (OR), 95% CI, and P values for cervical cancer incidence versus univariate and multivariate logistic regression analyses with other covariates.

Subgroup Variable	cancer=Cervical OR(95%CI)	P value
Age		
<65	0.90(0.86,0.94)	<0.01
≥65	0.91(0.85,0.98)	0.01
Race		
Non-Hispanic White	0.89(0.85,0.93)	<0.01
Non-Hispanic Black	0.88(0.80,0.97)	0.01
Mexican American	0.91(0.81,1.02)	0.11
Other Race	1.09(0.88,1.34)	0.43
Education level		
Less than high school	0.91(0.85,0.97)	<0.01
High school	0.95(0.891.02)	0.19
More than high school	0.88(0.83,0.92)	<0.01
Family PIR		
<2.3	0.94(0.90,0.98)	0.01
≥ 2.3	0.89(0.84,0.94)	<0.01
BMI		
<25	0.90(0.83,0.96)	<0.01
≥25	0.90(0.86,0.94)	<0.01
Smoking behavior		
never	0.90(0.84,0.96)	<0.01
former	0.88(0.80,0.95)	<0.01
now	0.92(0.86,0.98)	0.01
Alcohol consumption		
never	0.96(0.86,1.08)	0.51
former	0.95(0.88,1.03)	0.19
mild	0.93(0.82,0.98)	0.01
moderate	0.86(0.79,0.94)	<0.01
heavy	0.86(0.81,0.92)	<0.01
Energy intake		
<1580	0.90(0.85,0.97)	<0.01
≥1580	0.90(0.85,0.95)	<0.01
Hypertension		
yes	0.82(0.87,0.97)	<0.01
no	0.89(0.84,0.94)	<0.01
Diabetes		
yes	0.93(0.88,0.98)	0.01
no	0.90(0.86,0.94)	<0.01

PIR, energy intake is divided into groups according to median;model is a model adjusted for age, race, first menstruation age, living birth, education, BMI, PIR, smoking, alcohol consumption level, energy intake, hypertension and diabetes.

**Table 5:** Odds ratio (OR), 95% CI, and P values for ovarian cancer incidence versus univariate and multivariate logistic regression analyses with other covariates.

Subgroup Variable	cancer=Ovarian(95%CI)	P value
Age		
<65	0.89(0.84,0.94)	<0.01
≥65	0.95(0.87,1.03)	0.24
Race		
Non-Hispanic White	0.91(0.86,0.96)	<0.01
Non-Hispanic Black	0.93(0.82,1.05)	0.23
Mexican American	1.25(1.11,1.40)	<0.01
Other Hispanic	0.85(0.79,0.92)	<0.01
Other Race	0.91(0.86,0.96)	<0.01
Education level		
Less than high school	0.88(0.81,0.94)	<0.01
High school	0.91(0.86,0.96)	<0.01
More than high school	0.92(0.86,0.98)	0.01
Family PIR		
<2.3	0.87(0.79,0.96)	0.01
≥ 2.3	0.91(0.87,0.95)	<0.01
BMI		
<25	0.89(0.84,0.93)	<0.01
≥25	0.90(0.86,0.95)	<0.01
Smoking behavior		
never	0.91(0.87,0.96)	<0.01
former	0.79(0.64,0.98)	0.03
now	0.90(0.62,1.16)	0.40
Alcohol consumption		
never	1.01(0.87,1.17)	0.91
former	0.91(0.86,0.96)	<0.01
mild	0.86(0.78,0.95)	<0.01
moderate	0.90(0.82,0.99)	0.03
heavy	0.89(0.68,1.18)	0.43
Energy intake		
<1580	0.87(0.82,0.92)	<0.01
≥1580	0.93(0.87,0.99)	0.03
Hypertension		
yes	0.92(0.88,0.96)	<0.01
no	0.83(0.75,0.93)	<0.01
Diabetes		
yes	0.89(0.78,1.01)	0.06
no	0.90(0.87,0.94)	<0.01

PIR, energy intake is divided into groups according to median;model is a model adjusted for age, race, first menstruation age, living birth.

## Discussion

Changes before and after menopause and their relationship with gynecologic oncology are receiving increasing attention, which is also an important public health issue that has attracted the attention of researchers. Of the hormone levels caused by menopause, estrogen is undoubtedly the most affected (23, 24). Estrogen is a major female sex hormone that determines secondary sexual characteristics and influences the development and function of the female reproductive system. Changes in this hormone are associated with pathological menopausal syndromes such as sleep/mood disorders, vasodilatory symptoms (including hot flashes and night sweats), urogenital atrophy, bone loss and osteoporosis, psychiatric disorders, skin lesions, cardiovascular disease, metabolic disorders and obesity (25, 26). While endogenous estrogen protects a woman’s body before menopause, continued exposure to exogenous estrogen is a recognized risk factor for many types of cancer. Premature menopause is the premature cessation of estrogen production and ovulation by the ovaries, causing a woman to permanently stop menopause before age 40. Most premature menopause is caused by ovariectomy, or premature ovarian failure due to cancer treatments such as chemotherapy and radiation therapy, and is called “induced premature menopause” due to medical treatments such as surgery, chemotherapy, and radiation therapy before age 40. However, a small number of women with premature menopause who have not undergone ovarian surgery stop having cramps at a young age due to physical illness, genetics, emotional stress, or other unknown reasons, a condition known as “Primary Ovarian Insufficiency (POI)” (27–29). Studies have shown that hormonal and metabolic changes resulting from menopause are associated with cancer risk (30). According to a statistical analysis in the United States, the risk of invasive cancer increases exponentially after menopause. This is similar to our experimental findings that the younger the age of menopause, the higher the risk of gynecologic cancers in women. Cancer is now recognized as a metabolic disorder.The development of cancer may be related to premature hormonal changes in a woman’s body, which lead to disruption of the body’s metabolism (31, 32). This may be an important reason why menopause leads to the development of cancer. Previous studies have implicated estrogen in the development and progression of various cancers, including ovarian and endometrial cancers (33–35). In our study, we looked at whether age at menopause was associated with the incidence of gynaecological cancers, using age of menopause as a variable. The results confirmed that there was an inverse relationship between the age of menopause and the incidence of cervical and ovarian cancer, which provided an important scientific basis for universal screening of related cancers in the perimenopausal period and different menopausal age groups. Premature menopause has earlier and longer lasting hormonal changes in the body, which may be a trigger for cancer.

Estrogen is known to be associated with ovarian cancer (36). At the cellular level, estrogen binds to ERα leading to transcriptional activation of estrogen-responsive genes, which act on the signaling system for cell division and differentiation, among these genes are proto-oncogenes (such as c-fos, c-myc, and HER2/neu), cell cycle regulation cyclins, growth factors, etc. Membrane-bound G-protein-coupled estrogen receptor (GPER) binds to activate the second messenger system and promote tumor growth. In addition, the metabolic activation of estrogen produces free radicals that cause mutations, and the accumulation of various genetic mutations in the fallopian tubes and ovarian cells will lead to tumorigenic transformation of cells. The study by Appleby P et al. found that the relative risk of cervical cancer in people who took Combined estrogen–progestogen contraceptives (oral contraceptives or OCs) for ≥ 5 years was 1.9 times that of those who did not take it, and the incidence of cervical cancer was 10 years after ≥ withdrawal of the drug compared to that of those who did not take it (37). The carcinogenicity of estrogen and ER to the uterine cervix has been most strongly demonstrated in HPV transgenic mice. It was found that these transgenic rats were more likely to progress to cervical cancer if they were given estrogen (38–40). A large body of epidemiologic evidence suggests that endogenous and exogenous sex hormones, along with HPV infection, also affect a woman’s risk of developing the disease (41). In studies related to the development of cancer during menopause, age at menopause, as well as age at menarche, has been strongly associated with the risk of ovarian cancer in women. The increased risk of developing cervical cancer may be associated with ongoing estrogenic changes (27, 42, 43). Premature menopause is characterized by changes in various hormones in the female body, especially estrogen. When sustained changes in estrogen cause continuous beneficial or detrimental effects on the organism, it eventually leads to changes in the organism. Most importantly, some of the metabolic changes caused by estrogen have a significant impact on the development of cancer. There have been many studies on menopause, but they have not been adjusted or stratified for the potentially important variable of menopause (age). We have found that the incidence of gynecologic cancers varies by age at menopause, and that earlier menopause is associated with a higher risk of the gynecologic cancers ovarian and cervical cancer.

In our study, we found that age at menopause was negatively correlated with the incidence of ovarian and cervical cancer, and the earlier the age at menopause, the higher the prevalence, which may be related to the decrease in estrogen levels caused by menopause, and the earlier the menopause, the more fluctuating the changes in the body’s hormone levels, which leads to a series of changes in lipid metabolism, mood, and cardiovascular changes, and the alteration of lipid metabolism may play an important role in the process of cancer development. Studies have shown that lower estrogen levels lead to an accumulation of central adipose tissue and that increased central obesity is associated with an increased risk of cardiometabolic diseases, certain cancers, osteoarthritis and dementia (44, 45). In addition, estrogen deficiency increases the risk of cardiovascular disease. Renin-angiotensin system activation and impaired arterial endothelial function. Altered lipid metabolism leads to impaired metabolism in adipose tissue and plays a key role in the synthesis of excess fatty acids, adipocytokines, pro-inflammatory cytokines, and reactive oxygen species, which cause lipid peroxidation and contribute to the development of insulin resistance, abdominal obesity, and dyslipidemia, which may be correlated with the prevalence of cancers caused by menopause (46, 47). Studies have shown that the use of hormone therapy has been shown to mitigate some, although not all, of these risks. Individualized hormone therapy is important for women with early estrogen deficiency, and premenopausal women may require higher doses to approach physiological concentrations. It is also important to address the psychological impact of early menopause and to assess fertility options and the need for contraception when ovaries are intact (48–50). Age at menopause may influence the prevalence of ovarian and cervical cancer by affecting metabolism. Thus, we wanted to obtain information on the impact of age at menopause on future cardiovascular disease prevention, cancer screening, osteoporosis and fracture risk in women. In addition, it is important to realize the psychological impact of early menopause.

## Conclusion

Our study can bring some inspiration for clinical work. For example, for female patients at different menopausal ages who should receive different health management, we can perform early screening and use various methods to reduce the negative effects of menopause. Like most studies, our study has some drawbacks. First, there may be differences in the organismal changes that result from different menopausal ages. Second, another issue to consider is whether postmenopausal women develop diseases of the reproductive oncology system related to their life circumstances. Our results do not establish a causal relationship between age at menopause and common reproductive system tumors, but only an association between their correlation obtained by analysis of the study’s observational data. Finally, we did not consider other potential confounders, such as sleep quality, socioeconomic status, etc. Further studies with more variables and larger populations should be conducted to validate our results.

## Data availability statement

Publicly available datasets were analyzed in this study. This data can be found here: https://www.cdc.gov/nchs/nhanes/index.htm.

## Author contributions

GC and MW conceived of and drafted the manuscript. HS, JL, and YF collected the data. HL, YS, and YZ managed and cleaned the data. BZ criticized and revised the manuscript. All authors contributed to the article and approved the submitted version.
